# Zingerone Inhibits the Neutrophil Extracellular Trap Formation and Protects against Sepsis via Nrf2-Mediated ROS Inhibition

**DOI:** 10.1155/2022/3990607

**Published:** 2022-01-28

**Authors:** Yingjie Zhu, Dexiang Wang, Jingjing Luo, Jing Jie, Han Liu, Liping Peng, Xiaoxue Bai, Dan Li

**Affiliations:** ^1^Department of Respiratory Medicine, Center for Pathogen Biology and Infectious Diseases, Key Laboratory of Organ Regeneration and Transplantation of the Ministry of Education, The First Hospital of Jilin University, Changchun 130021, China; ^2^Department of Pulmonary and Critical Care Medicine, Qilu Hospital, Cheeloo College of Medicine, Shandong University, China; ^3^Department of General Practice, Center for Pathogen Biology and Infectious Diseases, Key Laboratory of Organ Regeneration and Transplantation of the Ministry of Education, The First Hospital of Jilin University, Changchun 130021, China

## Abstract

Neutrophils release chromatin and antimicrobial proteins to trap and kill microbes, which is termed as neutrophil extracellular trap (NET) formation. NETs play a pivotal role in host defense against infection. However, emerging evidence indicated that NETs also contribute to an exaggerated inflammatory response and organic injuries in sepsis. Zingerone, a natural compound extracted from *Zingiber officinale*, exerts antioxidant, anti-inflammatory, and antioncogenic properties. In this study, we found that treatment with zingerone reduced organ injury and improved the outcome in a cecal ligation puncture- (CLP-) induced polymicrobial sepsis model. Administration of zingerone also alleviates reactive oxygen species (ROS) accumulation and systematic inflammation in septic mice and inhibits neutrophil extracellular traps (NETs) formation *in vivo* and *in vitro*. Furthermore, inhibition of nuclear factor erythroid 2-related factor 2 (Nrf2) with its specific antagonist significantly counteracted the suppressive effects of zingerone on ROS and NETs and retarded the protective role of zingerone against sepsis-associated organ injury. In addition, exposure to zingerone does not affect phagocytic activity of neutrophils in vitro and bacterial dissemination in vivo. Above all, our results indicate that zingerone treatment obviously attenuates NET formation and inflammatory response via Nrf2-mediated ROS inhibition, thus providing a novel therapeutic strategy against sepsis-induced injury.

## 1. Introduction

Sepsis is a life-threatening syndrome characterized by excessive inflammatory responses from the host that causes multiple organ dysfunction [1]. About 18 million new cases of sepsis are diagnosed worldwide each year, and the incidence continues to increase dramatically [2]. Epidemiologic surveys show that the mortality from sepsis and septic shock is as high as 30–50% [3]. Antibiotics play a key role in the therapy of severe sepsis. Nevertheless, their serious side effects, such as antibiotic resistance, dysbacteriosis, and acute kidney injury, impelled us to seek new treatment strategies and medications [4, 5].

Polymorphonuclear neutrophil (PMN) granulocytes play an essential role in the innate immune response to infection. Prior studies have demonstrated that neutrophils eliminate infectious organisms by a combination of phagocytosis, degranulation, and release of NETs [6]. NETs are large, extracellular, and web-like DNA-containing structures, which consist of a chromatin backbone with attached antimicrobial proteins, such as histones, myeloperoxidase (MPO), and neutrophil elastase [7]. The NETs not only help neutrophils capture and kill pathogens efficiently but also destroy host tissues [8–12]. Overreleased NETs in lung fungal infections induce damage to alveolar epithelial cells. In addition, NETs can cause liver damage in sepsis caused by methicillin-resistant *Staphylococcus aureus* [13, 14]. Therefore, NET formation inhibition has been regarded as a potential therapeutic approach for infectious diseases [15, 16]. PMA-induced NET formation depended on reactive oxygen species (ROS) produced by NADPH oxidase [17]. And ROS mainly derived from mitochondria has been reported to be involved in NADPH oxidase activation and in the occurrence of NETosis caused by multiple stimuli [18]. Therefore, we hypothesize that the reduction of ROS contributes to the inhibition of NETs and prevention of organ damage from infections.

Ginger is a traditional Chinese medicine that has been widely employed for treatment of inflammatory disorders, the common cold, nausea, vomiting, and pain [19]. The active components extracted from ginger contain gingerols, shogaols, paradols, and zingerone (ZIN) [20]. It has been reported that ZIN possesses anti-inflammatory [21, 22] and antioxidant [23, 24] capacities in *in vitro* and *in vivo* experiments. Its potent pharmacological properties inspired us to explore the effects of ZIN in sepsis treatment.

In this current study, we found that ZIN significantly improved survival and clinical symptoms in polymicrobial sepsis. In addition, ZIN administration relieved organ damage, reduced ROS accumulation and systematic inflammation, and inhibited the NET formation. We also demonstrated the pharmacological properties of ZIN in sepsis are attributed to the activation of the nuclear factor erythroid 2-related factor 2 (Nrf2) pathway.

## 2. Materials and Methods

### 2.1. Reagents

Zingerone (>98% purity, CAS 122-48-5) was purchased from Herbpurify Co., Ltd. (Chengdu, China). Phorbol 12-myristate 13-acetate (PMA) was obtained from Sigma-Aldrich (USA). The Nrf2-specific inhibitor ML-385 was purchased from Selleck Chemicals (Shanghai, China).

### 2.2. Establishment of a Cecal Ligation Puncture- (CLP-) Induced Sepsis Model

Male C57 mice 6-8 weeks of age were obtained from Liaoning Changsheng Biotechnology Co., Ltd. (Liaoning, China) and maintained in animal facilities (20-25°C, 50-60% humidity, and 12 h light/12 h dark cycle with free access to sterile food and water) in accordance with Chinese legislation on the use and care of laboratory animals. Animal experiments were approved by the Animal Ethics Committee of the First Hospital of Jilin University.

The septic model was established by CLP procedure as previously described [25]. Briefly, mice were anesthetized by intraperitoneal injection of 1% pentobarbital (~120 *μ*l per 20 g), and then, the abdomen was shaved and disinfected. The abdominal cavity was carefully exposed, the cecum was identified, and 60% of its total length was ligated. A 27-gauge needle was used to puncture it. In addition, the control mice underwent a similar procedure of cecum mobilization without ligation and puncture. Mice were observed to assess the development of the following clinical symptoms for 24 h after CLP, as previously described [26]: piloerection, lethargy, tremor, periorbital exudates, respiratory distress, and diarrhea. Severe sepsis was defined as a clinical score > 3, and moderate sepsis was defined as a clinical score < 3. The mice were monitored for body temperature every 12 h for the first 24 h after CLP.

5 mice in each group were randomly sacrificed for histological analysis and NET evaluation in vivo 72 h after surgery, and 10 mice in each group were raised for survival analysis. And survival rates were determined in mice for 8 days.

First, the mice were randomly divided into following groups: control, CLP, CLP+zingerone 25 mg/kg body weight (CLP+ZIN25), CLP+zingerone 50 mg/kg (CLP+ZIN50), and zingerone 50 mg/kg (ZIN50). The animals in the last group were not submitted to CLP but were treated with ZIN (50 mg/kg) to evaluate the toxicity of ZIN in vivo. The animals from the CLP+ZIN25, CLP+ZIN50, and ZIN50 groups received an intraperitoneal (i.p.) injection of ZIN daily for 3 days before surgery to 4 days after surgery. The control group was injected with the same solvent intraperitoneally at the same time period. ZIN doses have been determined based on previous studies [27, 28].

Next, to investigate the roles of Nrf2 in therapeutic effects of ZIN against sepsis, ML385 (30 mg/kg), dissolved in PBS with 5% DMSO, was administered through an i.p. injection daily for 4 days before CLP to 3 days after CLP [29]. The mice in the vehicle group received the same solvent intraperitoneally at the same time period.

### 2.3. Determination of the Levels of Cytokines in Serum

After performing the CLP, blood samples (<30 *μ*l) were obtained at predetermined time points via tail vein puncture for serum level detection of inflammatory cytokines by enzyme-linked immunosorbent assays (ELISA). Interleukin- (IL-) 1*β*, IL-6, tumor necrosis factor- (TNF-) *α*, interferon- (IFN-) *γ* ELISA kits were used to detect the level of these cytokines according to the manufacturer's instructions. OD values were obtained using an ELISA plate reader by measuring absorbance at 450 nm wavelength.

### 2.4. Measurement of Oxidative Markers in Serum

Malondialdehyde (MDA), glutathione (GSH), and the Trolox equivalent antioxidant capacity (TEAC) in serum were determined by commercial kits (Beyotime) according to the manufacturer's protocol.

### 2.5. Colony-Forming Unit Assay

Bacterial loads were determined in peritoneal fluid and blood as previously described [30]. The peritoneal fluid and blood were collected from mice 48 h after CLP. 10 *μ*l of samples were diluted using sterile PBS and plated on LB agar plates.

### 2.6. Human Neutrophils Isolation and Ethics Statement

Ethical approval for collecting human peripheral blood was obtained from the Ethics Committee of the First Hospital of Jilin University. Human peripheral blood was collected from healthy volunteers in K2 EDTA-containing tubes. These volunteers all signed an informed consent form. Briefly, neutrophils were isolated by density gradient centrifugation using a peripheral blood neutrophil separation kit (Solarbio, Beijing, China) according to the manufacturer's instructions. The isolated neutrophils were washed in PBS and resuspended in appropriate amount of cold PBS before use.

### 2.7. The Isolation of Mouse Bone Marrow Neutrophils

The isolation of murine bone marrow neutrophils was performed as previously reported [31]. The mice were euthanized by dislocation, the tibia and femur were separated, and the connective tissue and muscle removed. Both ends of the bone were cut, and the bone marrow cavity was washed with 0.9% sodium chloride solution using a 1 ml syringe. The washing liquid was filtered in a sterile centrifuge tube and centrifuged at 270 g for 5 min to collect the cells. Then, the cells were resuspended in 3 ml 0.9% sodium chloride solution and placed in 9 ml Histopaque-1077, centrifuged at 2000 g without brake for 20 min at 4°C. The supernatant was discarded, and the cells were resuspended with 5 ml 0.9% sodium chloride solution, carefully placed in 10 ml Histopaque-1119 and again centrifuged. Subsequently, the intermediate flocculent layer was collected as neutrophils. Isolated neutrophils purity was examined by flow cytometry and reached >90%.

### 2.8. Histological Analysis

The lungs, kidneys, livers, and spleens of the mice were completely removed and fixed with 4% formaldehyde. The tissues were embedded in paraffin after fixing for 24 h. The sections were prepared for hematoxylin-eosin (HE) staining for examination under a light microscope. For lung injury scores, the lung injury characteristics (alveolar capillary congestion, hemorrhage, inflammatory cell infiltration, alveolar wall thickness, and hyaline membrane formation) were analyzed in 3 different lung slices using the following criteria: 0, absence of lesions (normal); 1–4, 10–40% (mild); 5–6, 50–60% (moderate); 7–8, 70–80% (severe); and 9–10, 90–100% (very severe) [32]. Liver injury scores were evaluated following the prior literature [33]. For kidney injury scores, the following lesions were evaluated: tubular dilation, brush border loss, tubular vacuole/necrosis, and cast formation. The percentage of these injuries was counted on a scale from 0 to 10: 0, absence of lesions (normal); 1–4, 10–40% (mild); 5–6, 50–60% (moderate); 7–8, 70–80% (severe); and 9–10, 90–100% (very severe) [32].

### 2.9. NET Observation In Vitro and In Vivo

Giemsa staining and immunofluorescence were used to observe NETs. Sterile glass sheets treated with polylysine were preplaced in 24-well plates, and neutrophils isolated from human peripheral blood were seeded at 2 × 10^5^ cells/well. Neutrophils were stimulated with PMA (100 nM) for 3 hours at 37°C. Then, 0.5 ml of 4% paraformaldehyde was added to each well and kept at 4°C overnight. The next day, the cell slides were carefully removed from the 24-well plate, washed with PBS, Giemsa-stained, and observed directly under an optical microscope. For immunofluorescence, the cell slides were permeabilized with PBS containing 0.5% Triton X-100 for 1 min. The blocking buffer, constituted by PBS containing 5% fetal bovine serum (FBS), was carefully added to the slides in a wet box at 37°C for 30 min. Subsequently, the slides were incubated with a mixture of primary antibody (anti-H3Cit) and blocking buffer in a wet box at 37°C for 1 h and then incubated with secondary antibody for 1 h. After 3 washes with PBS, the slides were DAPI-stained avoiding light for 10 min, washed with PBS twice, and visualized using a laser confocal microscope.

Immunofluorescence assays of the lung sections were also performed. Briefly, the lung sections were incubated overnight with primary antibody (anti-H3Cit) at 4°C after blocking with PBS containing 5% BSA. Then, FITC-tagged secondary antibodies were added to the sections for 1 h at room temperature. After 3 washes with PBS, the sections were DAPI-stained avoiding light for 15 min, washed with PBS twice and visualized using a laser confocal microscope.

### 2.10. Measurement of Reactive Oxygen Species (ROS)

The effect of ZIN on ROS generation in neutrophils was evaluated using DCFHDA assay kit (Beyotime, China). In brief, neutrophils under different conditions were treated with 20 *μ*M DCFHDA. Then, a flow cytometer (Beckman Coulter) was used for evaluating positive staining cells.

### 2.11. DNA Content Evaluation by SYTOX Green Staining

DNA content was measured by flow cytometry after SYTOX Green staining. Briefly, neutrophils treated under different conditions were washed twice with cold PBS and pelleted by centrifugation at 500 g for 5 min. Subsequently, they were incubated with 5 *μ*M SYTOX Green nucleic acid stain (Invitrogen, Carlsbad, CA, USA) at room temperature for 30 min. Then, the neutrophils were washed once with PBS and fixed with ethanol. A FACS flow cytometer (Beckman Coulter, Fullerton, CA) was used to analyze positive staining cells.

### 2.12. Myeloperoxidase (MPO)/DNA Detection

An ELISA kit that captures DNA-associated MPO was used to quantify NETs. Samples were obtained from the supernatants of treated neutrophils under different conditions or the supernatants of bronchoalveolar lavage fluid (BALF) in different groups of mice. 96-well plates were coated with anti-MPO Ab (Invitrogen, Carlsbad, CA, USA) overnight at 4°C. After washing with cold PBS, samples (20 *μ*l) were added to the wells by mixing with 80 *μ*l incubation buffer containing a peroxidase-labeled anti-DNA antibody (Cell Death ELISA PLUS, Roche). The plate was incubated and gently shaken for 2 hours at room temperature. Then, 100 *μ*l peroxidase substrate (ABTS) was added and absorbance was read at 405 nm wavelength.

### 2.13. Cell-Free DNA (cfDNA) Detection

Serum cfDNA was measured with M200 Pro (Tecan, Switzerland) using PicoGreen (Invitrogen). The DNA content was calculated from a standard curve generated from standard samples.

### 2.14. Evaluation of Neutrophil Phagocytic Function

Bacteria (*Klebsiella pneumoniae*) were opsonized with 50% autologous normal human serum at 37°C for 30 minutes. The opsonized bacteria were washed with sterile PBS, and then, the bacteria resuspended in the RPMI/H medium. In order to determine whether ZIN can enhance the bactericidal capacity of neutrophils, polymorphonuclear neutrophils (PMNs, 2^∗^10^6^ cells/ml) were incubated with ZIN at 37°C for 30 minutes. Then, the bacteria were added to the ZIN pretreated neutrophils at an infection multiplicity of 1 : 1, and the samples were incubated at 37°C for a period of time. Subsequently, smear samples were stained with Giemsa and observed under an optical microscope to calculate the phagocytosis percentage and neutrophil phagocytosis index in order to determine the neutrophil phagocytic function.

### 2.15. Western Blot Analysis

The total proteins of the neutrophils from human or mice were lysed in RIPA Buffer, and the BCA assay (Beyotime, Shanghai, China) was used to measure the protein concentration and ensure that the protein loading was the same in all SDS-PAGE gel wells. A total of 40 *μ*g protein was separated by 10% SDS-PAGE and transferred to polyvinylidene fluoride membranes. The membranes were incubated with primary antibodies (phosphorylated- (p-) Nrf2, Lamin B, and GAPDH (ABclonal, Wuhan, China). The next day, the membrane was incubated with horseradish peroxidase- (HRP-) labeled secondary antibodies (ABclonal, Wuhan, China) at room temperature for 2 h. Finally, the protein bands on the membranes were detected by with enhanced chemiluminescence detection reagents.

### 2.16. Statistical Analysis

All data were plotted and analyzed using the GraphPad Prism 6 (La Jolla, CA). The differences between the data sets were analyzed with one- or two-way analysis of variance (ANOVA). Mouse survival rates were calculated using Kaplan–Meier curves. *P* values < 0.05 were considered indicative of statistical significance.

## 3. Results

### 3.1. ZIN Treatment Attenuated CLP-Induced Polymicrobial Sepsis in Mice

**Figure 1:**
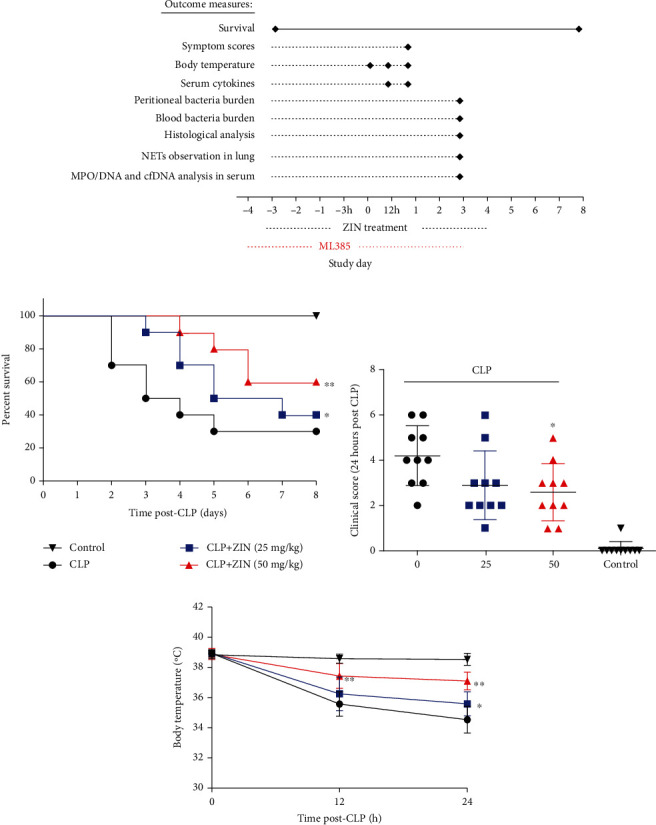
ZIN treatment attenuated CLP-induced polymicrobial sepsis in mice. (a) Experimental procedures. (b) Survival rate was monitored for 8 days. Mortality rates were compared using the Kaplan–Meier method with the Log-rank test. (c) Mice were scored for 6 different signs of sepsis for 24 h after CLP. Clinical score > 3 was defined as severe sepsis. (d) The body temperature of the mice in different groups was monitored at 12 and 24 hours after CLP. ^∗^*P* < 0.05 versus the CLP group; ^∗∗^*P* < 0.01 versus CLP group.

To evaluate the ZIN effects on sepsis, a mid-grade CLP-induced sepsis model was established in mice, as described previously [25].  Figure 1(a) illustrates the experimental procedures for establishing this model. The mouse survival rates were approximately 70% at the end of day 8 after performing the CLP (Figure 1(b)). However, ZIN pretreatment increased the survival rates of sepsis mice. Moreover, the symptoms of murine sepsis were assessed with clinical score (Figure 1(c)). At 24 h post-CLP, 90% of mice in the CLP group exhibited severe or moderate sepsis (mice with a clinical score ≥ 3). After ZIN pretreatment, there was a significant decrease of mice exhibited severe or moderate symptoms. Then, the body temperature in the CLP group was significantly lower than that of the control group and the body temperature was significantly higher in the ZIN-treated mice compared to the model group (Figure 1(d)). These results indicated that ZIN pretreatment increased survival rates and alleviated disease progression in CLP-induced sepsis.

### 3.2. ZIN Diminished the Systematic Inflammation Response and Oxidative Stress in Sepsis Mouse Model

**Figure 2:**
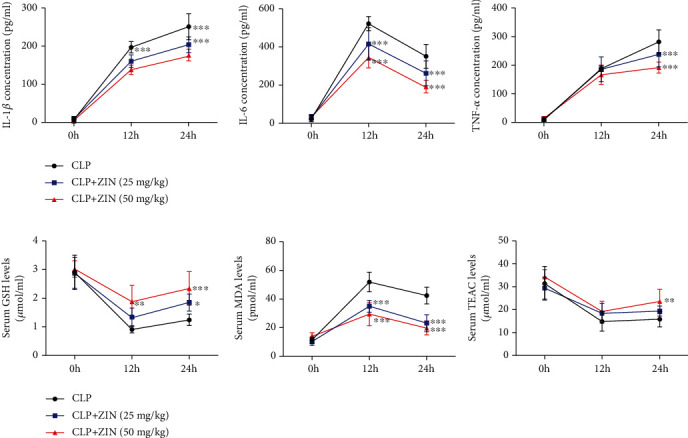
ZIN diminished the systematic inflammation response and oxidative stress in the septic mouse model. (a) Detection of cytokine secretion (IL-1*β*, IL-6, and TNF-*α*) as well as (b) MDA, GSH, and TEAC in mouse serum from 12 h and 24 h post-CLP. ^∗^*P* < 0.05 versus the CLP group; ^∗∗^*P* < 0.01 versus the CLP group; and ^∗∗∗^*P* ≤ 0.001 versus the CLP group.

The systemic inflammatory response is associated with mortality from sepsis. Therefore, cytokine secretion in serum 12 h and 24 h post-CLP was measured. Several proinflammatory cytokines (IL-1*β*, IL-6, and TNF-*α*) were observed to be increased in CLP mice. The cytokine IL-1*β* is an important component that requires the activation of the NF-*κ*B pathway. ZIN administration significantly suppressed the IL-1*β* production compared to the CLP group (Figure 2(a)). TNF-*α* plays a key role in sepsis occurrence and development. A significant reduction in serum TNF-*α* concentration was observed in ZIN-treated mice compared to the untreated group (Figure 2(a)). The concentrations of IL-6 showed a clear tendency to increase after 12 h after CLP but gradually decreased over time. ZIN treatment suppressed IL-6 concentrations (Figures 2(a)). Aberrant accumulation of ROS is another feature of sepsis, which can aggravate inflammatory response and directly cause the cellular injury [34]. The levels of MDA, GSH, and TEAC were determined after ZIN treatment in septic mice, and the results showed that ZIN significantly reduced the levels of MDA and upregulated the levels of GSH and TEAC in a dose-dependent manner (Figures 2(b)). These data indicate ZIN treatment alleviates the systematic inflammation and oxidative stress in sepsis.

### 3.3. The Effects of ZIN on Organ Injuries in CLP-Induced Sepsis Mice

**Figure 3:**
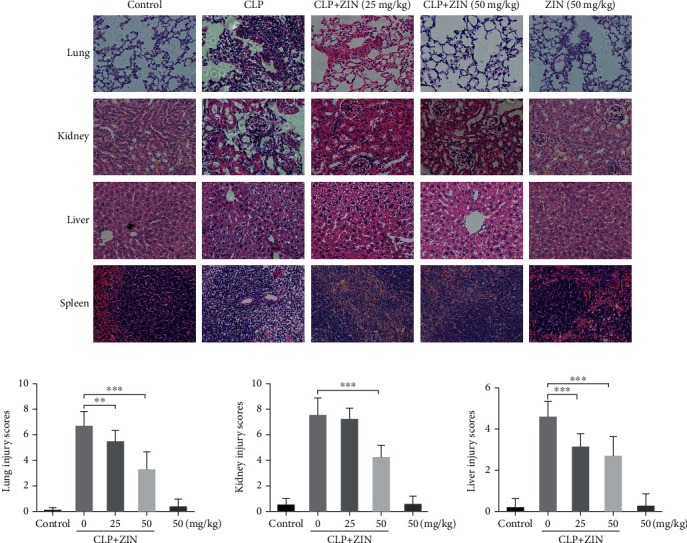
The effects of zingerone (ZIN) on organ injuries in CLP-induced sepsis mice. (a) H&E staining of lung, kidney, liver and spleen tissues performed 72 h post-CLP (light microscope, magnification ×400). (b) Lung, kidney, and liver injury scores were determined in each group. ^∗^*P* ≤ 0.05, ^∗∗^*P* ≤ 0.01, and ^∗∗∗^*P* ≤ 0.001.

Multiorgan dysfunction is the leading cause of death in sepsis. To verify the ZIN protective effect on distant organs in a sepsis model, histological analysis of the lung, kidney, liver, and spleen tissues were performed using H&E staining. ZIN treatment reduced lung injury, as evidenced by reduced inflammatory cell infiltration, hemorrhage, and interstitial edema (Figure 3). Moreover, CLP-induced sepsis mice exhibited severe liver and kidney tissue damage; however, ZIN treatment reversed this effect. Spleen sections in the CLP group showed spleen congestion accompanied by obvious white pulp consumption, and ZIN treatment also mitigated this effect. In addition, there was no damage to these organs in ZIN-treated (50 mg/kg) healthy mice not submitted to CLP. Taken together, these data revealed that ZIN treatment alleviated sepsis-induced organ damage.

### 3.4. ZIN Inhibited PMA-Induced ROS Production and the Formation of NETs through Nrf2 Pathway

**Figure 4:**
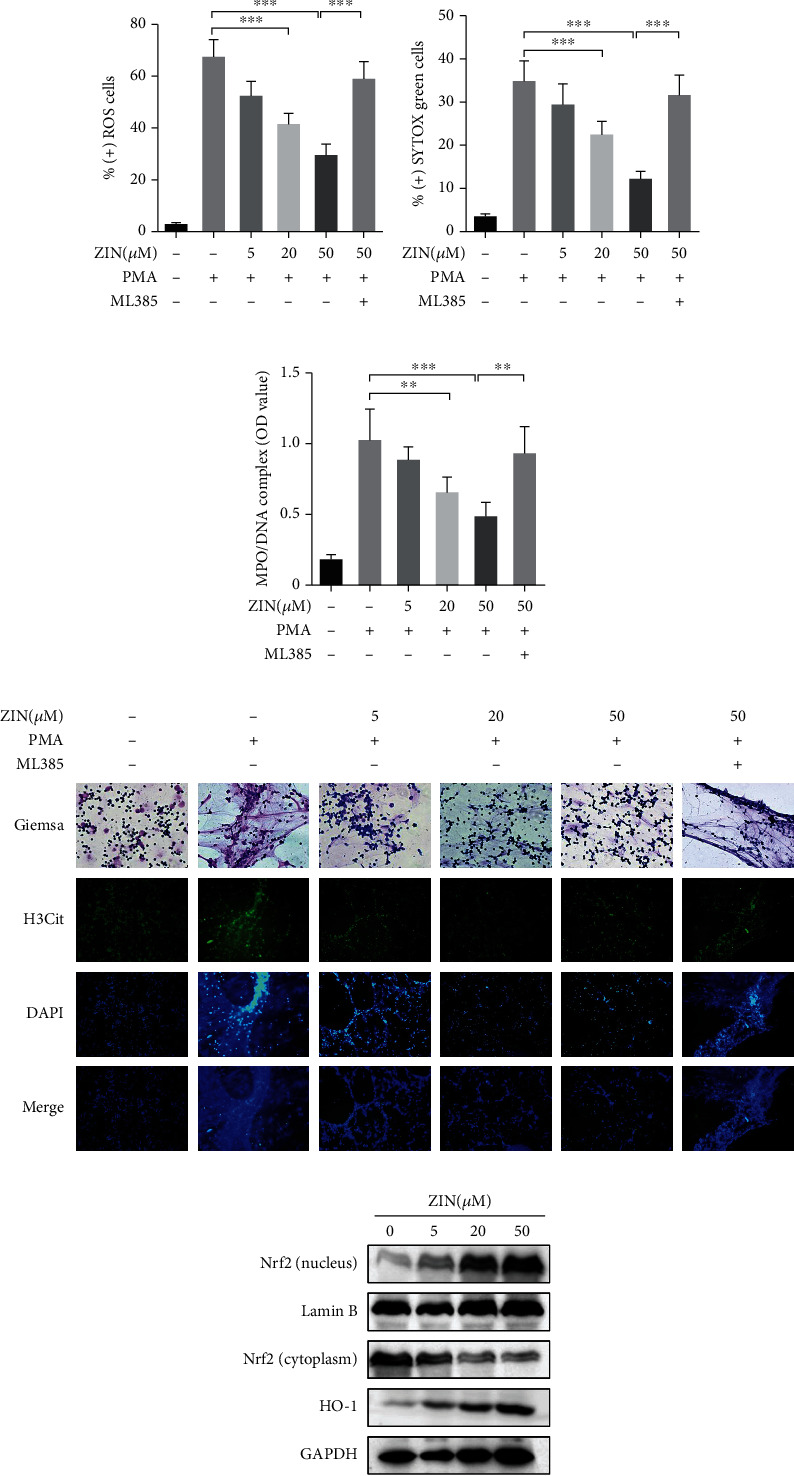
ZIN inhibited PMA-induced ROS production and the formation of NETs through Nrf2 pathway. (a) DCFH-DA staining and (b) SYTOX green staining of neutrophils in different groups was analyzed by flow cytometry. (c) ZIN effects on the MPO-DNA complex in neutrophil supernatant. (d) The effects of ZIN on NET production in human neutrophils stimulated with PMA for 3 hours (histone: green; dsDNA: blue) (fluorescence and light microscope, magnification ×200). (e) The effects of ZIN on activation of Nrf2/HO-1 signaling in neutrophils. ^∗^*P* ≤ 0.05, ^∗∗^*P* ≤ 0.01, and ^∗∗∗^*P* ≤ 0.001.

To evaluate the relationship between ZIN and ROS in vitro, neutrophils from the different treatments were prepared for flow cytometric analysis of DNA content using DCFH-DA staining. The results showed that ZIN pretreatment mitigated ROS production induced by PMA (Figure 4(a)). SYTOX green-positive green cells indicated the release of extracellular DNA (NETs). The results demonstrated that the positive rate for SYTOX green in the PMA group was relatively higher than in the control group. However, this trend was reversed by ZIN treatment in a dose-dependent manner (Figure 4(b)). PMA-induced MPO/DNA complex levels in neutrophils also showed the same trend (Figure 4(c)). We also evaluated the NET formation in each group via Giemsa and immunofluorescence staining. The results showed that ZIN pretreatment significantly suppressed NET release compared to neutrophils stimulated only with PMA (Figure 4(d)). Intriguingly, pretreatment with ML385, a specific inhibitor of Nrf2, remarkably abolished the inhibitory effects of ZIN on PMA-induced ROS production and NET formation (Figures 4(a)–4(d)), suggesting Nrf2 signaling contributed to pharmacological properties of ZIN.

We have previously reported that ZIN inhibits oxidative stress in airway epithelial cells via activation of Nrf2 pathway, thus protecting mice from bronchial asthma [24]. Therefore, the influence of ZIN on the Nrf2 pathway was assessed in neutrophils via Western blotting. As shown in Figure 4(e), the levels of the nuclear Nrf2 and HO-1 were markedly increased after ZIN stimulation, while the cytoplasmic Nrf2 levels were decreased. In summary, ZIN treatment significantly inhibited ROS production and NET formation via activation of the Nrf2 signaling pathway in a dose-dependent manner.

### 3.5. ZIN Inhibited NET Formation and Promoted the Nuclear Translocation of Nrf2 In Vivo

**Figure 5:**
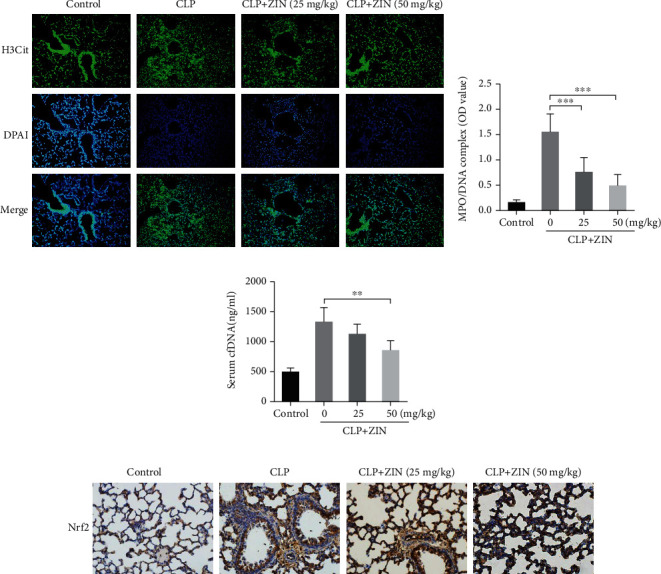
ZIN inhibited NETs formation and promoted the nuclear translocation of Nrf2 in vivo. (a) The effects of ZIN on NETs production in the lungs of CLP-induced septic mice (histone: green; dsDNA: blue) (fluorescence microscope, magnification ×400). ZIN effects on the levels of MPO-DNA complex in BALF supernatants (b) and cfDNA in plasma (c). (d) Representative immunohistochemical pictures of Nrf2 expression in lung sections from different groups (light microscope, magnification ×400). ^∗^*P* ≤ 0.05, ^∗∗^*P* ≤ 0.01, and ^∗∗∗^*P* ≤ 0.001.

In vivo studies showed that the lung sections of mice in the CLP group mice contained a higher level of histones compared to the ZIN pretreatment group. These studies also revealed that ZIN treatment decreased the neutrophil infiltration into the lungs of septic mice (Figure 5(a)) and MPO/DNA complex level in BALF supernatants was decreased after ZIN pretreatment in a dose-dependent manner (Figure 5(b)). cfDNA is the fundamental component of NETs. Serum cfDNA levels were significantly higher in the CLP group compared to the CLP+ZIN groups (Figure 5(c)). These results directly reflected that ZIN reduced the NET production in vivo.

Then, lung sections were immunohistochemically stained by the Nrf2 antibody to evaluate the effect of ZIN on Nrf2 expression in vivo. As shown in Figure 5(d), ZIN treatment promoted the nuclear translocation of Nrf2 in the lung of septic mice.

### 3.6. Inhibition of Nrf2 Retarded the Protective Role of Zingerone against Sepsis

**Figure 6:**
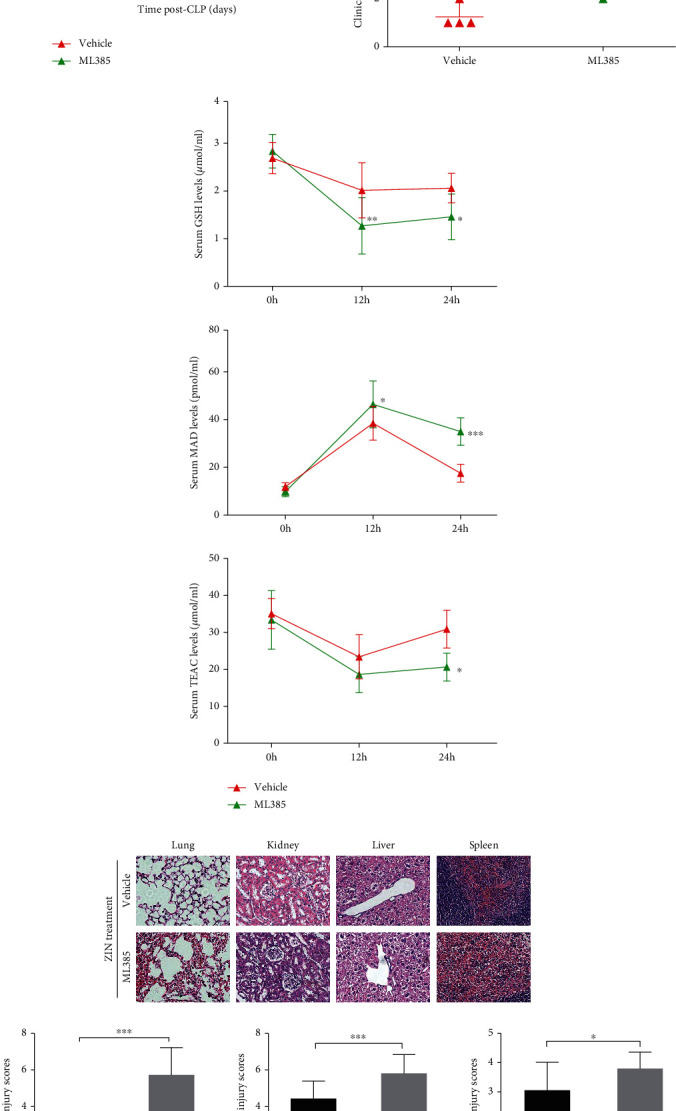
Inhibition of Nrf2 retarded the protective role of zingerone against sepsis. CLP induced septic mice were pretreated with ML385 (30 mg/kg), followed by treatment with ZIN (50 mg/kg). (a) Survival rate was monitored for 8 days. Mortality rates were compared using the Kaplan–Meier method with the Log-rank test. (b) Mice were scored for 6 different signs of sepsis for 24 h after CLP. Clinical score > 3 was defined as severe sepsis. (c) MDA, GSH, and TEAC in mouse serum from the vehicle or ML385 group. (d) H&E staining of the lung, kidney, liver and spleen tissues (light microscope, magnification ×400) and the injury scores corresponding to the organ damage. ^∗^*P* ≤ 0.05, ^∗∗^*P* ≤ 0.01, and ^∗∗∗^*P* ≤ 0.001 versus septic mice pretreated with vehicle.

To further confirm Nrf2 signaling plays a role in therapeutic effects of MA, ML385 was given prior to ZIN treatment in septic mice. As shown in Figure 6(a), pretreatment with ML385 remarkably decreased the survival rate of septic mice. In addition, the clinical score of ZIN-treated septic mice were increased in response to ML385 pretreatment (Figure 6(d)). For the oxidative stress-related indicators, pretreatment with ML385 increased the levels of MDA, as well as decrease the levels of GSH and TEAC (Figure 6(c)). By HE staining, we also found that ML385 obviously retarded the protective role of ZIN in sepsis-associated organs injury (Figure 6(d)). These data indicated that Nrf2 contributed to the therapeutic effects of ZIN *in vivo*.

### 3.7. ZIN Did Not Affect Neutrophil Bactericidal Activity In Vitro and Bacteria Dissemination In Vivo

**Figure 7:**
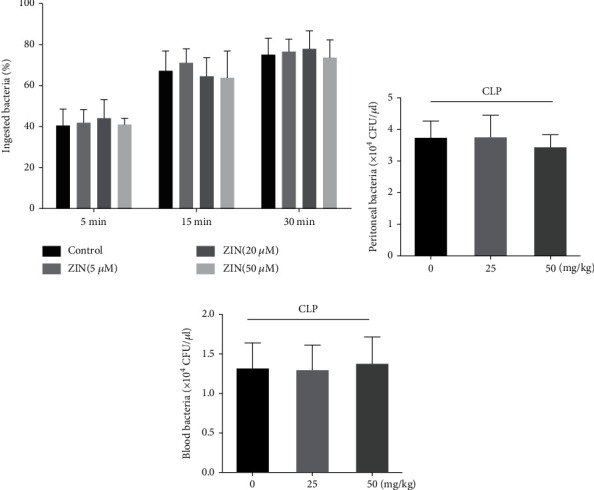
Zingerone (ZIN) did not affect neutrophil bactericidal activity in vitro and bacteria dissemination in vivo. (a) Detection of phagocytic ability of neutrophils stimulated with PMA and ZIN at different time points. (b, c) Colony-forming unit (CFU) analysis of the peritoneal lavage and the blood of mice from different groups.

To determine whether ZIN can increase the PMN bactericidal activity in vitro, neutrophils and *Klebsiella pneumoniae* were incubated for different times and Giemsa staining was used to evaluate the phagocytic capacity of neutrophils. ZIN-treated neutrophils showed no differences in the phagocyte percentage compared to the control group (Figure 7(a)). Previous studies have shown that NETs can restrict the dissemination of *Staphylococcus aureus*, *Salmonella typhimurium*, *Streptococcus pneumoniae*, and *Shigella flexneri* [35–38]. Thus, ZIN's ability to inhibit the spread of bacteria in sepsis mice was investigated. 72 hours after CLP, the peritoneal lavage and the blood of mice were collected and cultured for colony-forming units (CFU) analysis. ZIN-pretreated mice had no significant difference in peritoneal or blood bacterial loads compared with the control group (Figures 7(b) and 7(c)). Collectively, these data suggested that ZIN has no effect on PMN bactericidal activity in vitro and bacteria dissemination in vivo.

## 4. Discussion

ZIN has been previously demonstrated to exert antioxidant and anti-inflammatory effects.

We previously reported that ZIN treatment ameliorates airway inflammation and protects the OVA-induced oxidative stress in asthma by activating the Nrf2/HO-1 signaling pathways [24]. Also, as reported by Mani et al., ZIN played a beneficial role in mouse model of alcoholic hepatitis via reducing the oxidative stress and inflammation [39]. Similarly, ZIN attenuated vancomycin-induced hepatotoxicity in rats with the same mechanism [40]. ZIN also showed the antioxidant and anti-inflammatory effects in multiple disease models [41–43]. Nevertheless, this is the first study reporting the positive role of ZIN on the polymicrobial sepsis model. Our study showed that ZIN can alleviate clinical symptoms and sepsis-related organ damage and improved the overall survival rate in murine sepsis. We also demonstrated that ZIN treatment can inhibit the formation of NETs by Nrf2-mediated ROS inhibition in PMA-stimulated neutrophils. In addition, exposure to ZIN does not affect the phagocytic activity of neutrophils in vitro and the bacterial dissemination in vivo.

In the present study, we established a CLP-based sepsis model. It is considered the most representative model that can replicate the course of human polymicrobial sepsis [44–46]. Here, we observed that CLP caused a mortality rate close to 70% eight days after surgery. However, ZIN treatment significantly decreased the lethality rate and mitigated the disease symptoms, as well as maintained body temperature stable during the first 24 h after CLP. Correction of hypothermia during sepsis has been reported to have a positive prognostic impact by inhibiting IL-6 release [47]. The main pathological feature of sepsis is uncontrolled infection with high levels of plasma inflammatory factors and inappropriate leukocyte accumulation, resulting in multiple organ failure [48–50]. Therefore, we measured the level of various proinflammatory cytokines in septic mouse serum in the first 24 hours after CLP. The results showed that ZIN treatment attenuated systemic inflammation during sepsis. The occurrence of multiple organ failure appears to be directly associated with higher mortality among septic patients [51]. Thus, we performed histological analysis of major organs of mice in different treatment groups. We found that ZIN treatment mitigated organ damage in sepsis. Taken together, these data show that ZIN has strong protective effects on sepsis. So the question is, what molecular mechanisms could explain the ZIN protective role in the sepsis murine model?

Accumulating evidence has shown that NETs are closely related to occurrence and development of inflammatory diseases [6, 52–54]. A previous study has reported that NET digestion decreased systemic inflammation and organ damage [55]. It also has been reported that inhibiting the NET formation can be a potential therapeutic strategy for the treatment of pediatric sepsis [56]. In the present study, we observed that ZIN significantly inhibited the NET formation in PMA-stimulated neutrophils stimulated and in the septic mouse lungs in a dose-dependent manner. NETs can induce cytokine production (IL-1*β*, TNF-*α*, and IL-6) by activating macrophages and dendritic cells [57]. This may explain why ZIN can mitigate systemic inflammation during sepsis.

Moreover, aberrant ROS accumulation has been reported to participate in NET formation [58]. Thus, the levels of MDA, GSH, and TEAC were measured in PMA-stimulated neutrophils at different concentrations of ZIN and we found that ZIN significantly inhibited oxidative stress. Nrf2 signaling plays a pivotal role in modulating the redox hemostasis, which is targeted by a large cohort of antioxidant natural compounds [59–61]. Previously, we reported that ZIN activates the Nrf2 signaling pathway in airway epithelial cells, contributing to its antiasthmatic effects in mice [24]. In this study, we found ZIN treatment can also induce Nrf2 activation and the downregulation of ROS in neutrophils, suggesting the inhibitory effects of ZIN on NETs is due to Nrf2-dependent ROS inhibition.

Besides to their involvement in the formation of NETs, neutrophils proved to be a first line of defense against pathogens, presenting an important phagocytosis capacity. In brief, the fusion of the phagosome and intracellular granules induces the phagosome maturation. Then, the encapsulated bacteria will be attacked by a mixture of toxic molecules, including antibacterial proteins and strong oxidants [62]. Therefore, in this study, we evaluated the ZIN treatment effects on the phagocytic activity of neutrophils. The results demonstrated that ZIN had no effect on PMN bactericidal activity in vitro and on bacteria dissemination *in vivo*.

Collectively, these findings showed that ZIN exerted a protective role by inhibition of NET formation on the CLP-induced polymicrobial sepsis murine model. This protective mechanism of ZIN mainly relied on inhibiting the ROS via activation of Nrf2, rather than affecting the phagocytic ability of host PMN or the bacteria dissemination in vivo.

## Data Availability

Data availability may be granted by contacting the corresponding author.
